# Prolonged restraint stressor exposure in outbred CD-1 mice impacts microbiota, colonic inflammation, and short chain fatty acids

**DOI:** 10.1371/journal.pone.0196961

**Published:** 2018-05-09

**Authors:** Ross M. Maltz, Jeremy Keirsey, Sandra C. Kim, Amy R. Mackos, Raad Z. Gharaibeh, Cathy C. Moore, Jinyu Xu, Vasudevan Bakthavatchalu, Arpad Somogyi, Michael T. Bailey

**Affiliations:** 1 Pediatric Gastroenterology, Nationwide Children's Hospital, Columbus, OH, United States of America; 2 Center for Microbial Pathogenesis, The Research Institute, Nationwide Children’s Hospital, Columbus, OH, United States of America; 3 Department of Pediatrics, The Ohio State University College of Medicine, Columbus, OH, United States of America; 4 Campus Chemical Instrumentation Center Mass Spec and Proteomics, The Ohio State University, Columbus, OH, United States of America; 5 Department of Pediatrics, Division of Gastroenterology, Hepatology and Nutrition, University of Pittsburgh School of Medicine, Pittsburgh, PA, United States of America; 6 Department of Bioinformatics and Genomics, University of North Carolina at Charlotte, Charlotte, NC, United States of America; 7 Bioinformatics Services Division, Department of Bioinformatics and Genomics, University of North Carolina at Charlotte, Kannapolis, NC, United States of America; 8 The Division of Comparative Medicine, Massachusetts Institute of Technology, Cambridge, MA, United States of America; "INSERM", FRANCE

## Abstract

Stressor-exposure has been shown to exacerbate inflammation and change the composition of the gastrointestinal microbiota; however stressor-induced effects on microbiota-derived metabolites and their receptors are unknown. Thus, bacterial-produced short chain fatty acids (SCFAs), as well as microbial community composition, were assessed in the colons of mice exposed to stress during infection with *Citrobacter rodentium*. Mice were exposed to overnight restraint on 7 consecutive nights, or left undisturbed as a control. After the first exposure of restraint, mice were orally challenged with *C*. *rodentium* or with vehicle. Microbial community composition was assessed using 16S rRNA gene sequencing and SCFA levels measured using gas chromatography-mass spectrometry (GC-MS). Pathogen levels and colonic inflammation were also assessed 6 days post-infection. Results demonstrated that the microbial community structure and SCFA production were significantly affected by both stressor exposure and *C*. *rodentium*-infection. Exposure to prolonged restraint in the absence of infection significantly reduced SCFAs (acetic acid, butyric acid, and propionic acid). Multiple bacterial taxa were affected by stressor exposure, with the relative abundance of *Lactobacillus* being significantly reduced and directly correlated with propionic acid. *Lactobacillus* abundances were inversely correlated with colonic inflammation, supporting the contention that *Lactobacillus* helps to regulate mucosal inflammatory responses. Our data indicates that restraint stressor can have significant effects on pathogen-induced colonic inflammation and suggest that stressor-induced changes in the microbiota, microbial-produced SCFAs and their receptors may be involved.

## Introduction

Exposure to stressful situations disrupts homeostatic interactions between the host and its gut microbiota. This has been well demonstrated in laboratory animals where exposure to stress has been shown to both change the composition of the gut microbiota and to exacerbate experimental colonic inflammation [1]. For example, our lab has previously shown that stressor exposure prior to or during colonic infection increases susceptibility to the pathogen and worsens the severity of colonic inflammation [2–4]. Stressor-induced exacerbations of colonic inflammation also occur in mice administered dextran sulfate sodium (DSS) to induce colitis [5–7]. These findings in laboratory animals are consistent with clinical observations; stressor exposure has been associated with exaggerated inflammatory diseases in humans. This is well-illustrated in patients with inflammatory bowel disease whose disease severity can be related to exposure to perceived stress, with stress often being associated with a flare of one’s disease [8, 9].

The complete set of factors that contribute to exacerbation of the colonic immune response during stressful periods are not yet known, however gut microbiota are thought to be involved. The gut microbiota includes viruses, fungi, yeasts, protozoa, archaea, and bacteria that are found in the lumen of the intestines or associated with mucosal tissue. Humans are colonized by hundreds of different species of bacteria with the largest number of bacteria in the distal ileum and colon. In total, there are as many bacteria in the human body as there are human cells [10–14]. The commensal microbiota can significantly affect host physiology, with recent evidence demonstrating significant interactions between the gut microbiota and the host nervous system (i.e., the so-called brain-gut microbiota axis). A number of animal studies now indicate that activation of the nervous system (such as through exposure to experimental stressors) changes the composition of the luminal and mucosa-associated gut microbiota [2, 15–18]. For example, restraint (RST) stressor, which is a prolonged stressor, and social disruption (SDR), which is a social stressor, affect measures of alpha diversity (used to assess microbial diversity within a sample) and beta diversity (used to assess microbial diversity between samples) [2, 19]. These changes in the colonic microbiota are biologically meaningful and directly alter mucosal immune responses upon pathogen challenge. This was evident when germ-free mice that were colonized with stool from stressor-exposed mice had an increased inflammatory response to the pathogen *Citrobacter rodentium* in comparison to mice that were colonized with stool from un-stressed mice prior to pathogen challenge [4]. In addition, exposure to different types of stressors reduces the relative abundance of *Lactobacillus* [16, 20–22]. The probiotic *Lactobacillus reuteri* has been shown to alleviate the effects of stress on the colonic inflammatory response during infection in mice [23]. These studies demonstrate that stressor-induced changes in the microbiota affect the mucosal immune response to colonic pathogens.

The mechanisms by which stressor-induced changes in the microbiota influence the immune system are not well understood, but it is suspected that microbiota-produced metabolites from undigested foods influence the immune system. Thus far, no one has assessed whether exposure to a RST stressor affects the production of microbial metabolites. Short chain fatty acids (SCFAs), including acetic acid, propionic acid, and butyric acid, are produced by the commensal microbiota from indigestible carbohydrates and affect the immune system through multiple mechanisms, including up-regulation of T regulatory (T-reg) cells [24, 25]. SCFAs can act on the immune system or the enteric nervous system via inhibition of histone deacetylases that regulate gene expression and via the activation of G-protein coupled receptors (e.g., GPR41, GPR43, and GPR109A). G-protein coupled receptors are expressed on multiple cells types and found on many different tissues where they exhibit a variety of functions [24, 26–32]. GPR41 is found on enteric neurons, whereas GPR43 is found on immune cells in the lamina propria, T-reg cells, mucosal mast cells and neutrophils [26, 28, 31]. GPR41 and GPR43 receptors have been shown to be essential for inducing a Th1 acute inflammatory response because when mice are deficient in GPR41 and GPR43 in the intestinal epithelium, mice are not able to mount an inflammatory response to 2,4,6 trinitrobenzene sulfonic acid (TNBS) or to *C*. *rodentium* pathogen challenge [27, 29, 30]. Activation of GPR109A reduces intestinal inflammation in mice and humans. This anti-inflammatory activity is suspected to occur via alterations in the commensal gut microbiota [32–35].

The purpose of this study was to assess the effects of a prolonged RST stressor on microbial-produced SCFAs in outbred CD-1 male mice challenged with a colonic infection. The prolonged RST stressor induces the perception of confinement [36] and activates the autonomic nervous system and the hypothalamic-pituitary-adrenal axis [37, 38]. We focused on the production of SCFAs in the colon, which are exclusively produced by gut microbes and can significantly impact host mucosal immune responses. Thus, stressor-induced changes in SCFAs might be one way in which alterations in the microbiota impact colonic inflammation. This study aimed to determine if a prolonged restraint stressor decreases SCFA levels during infection to further understand microbiome changes in animals with stressor-enhanced colonic inflammation.

## Methods

### Animals

Male CD-1 mice, 6–8 weeks of age, were purchased from Charles River Laboratories (Kingston, NY). They were housed in groups of 3 per cage and kept on a 12 hr light:dark schedule with lights on at 0600. Mice were acclimated to the animal vivarium for a minimum of 7 days prior to experimentation. Standard chow and water were available *ad libitum* except during experimental procedures. All experimental procedures were approved by the Ohio State University Animal Care and Use Committee. All methods were performed in accordance with The Ohio State University Animal Care and Use Committee.

### Experimental design

**Fig 1:**
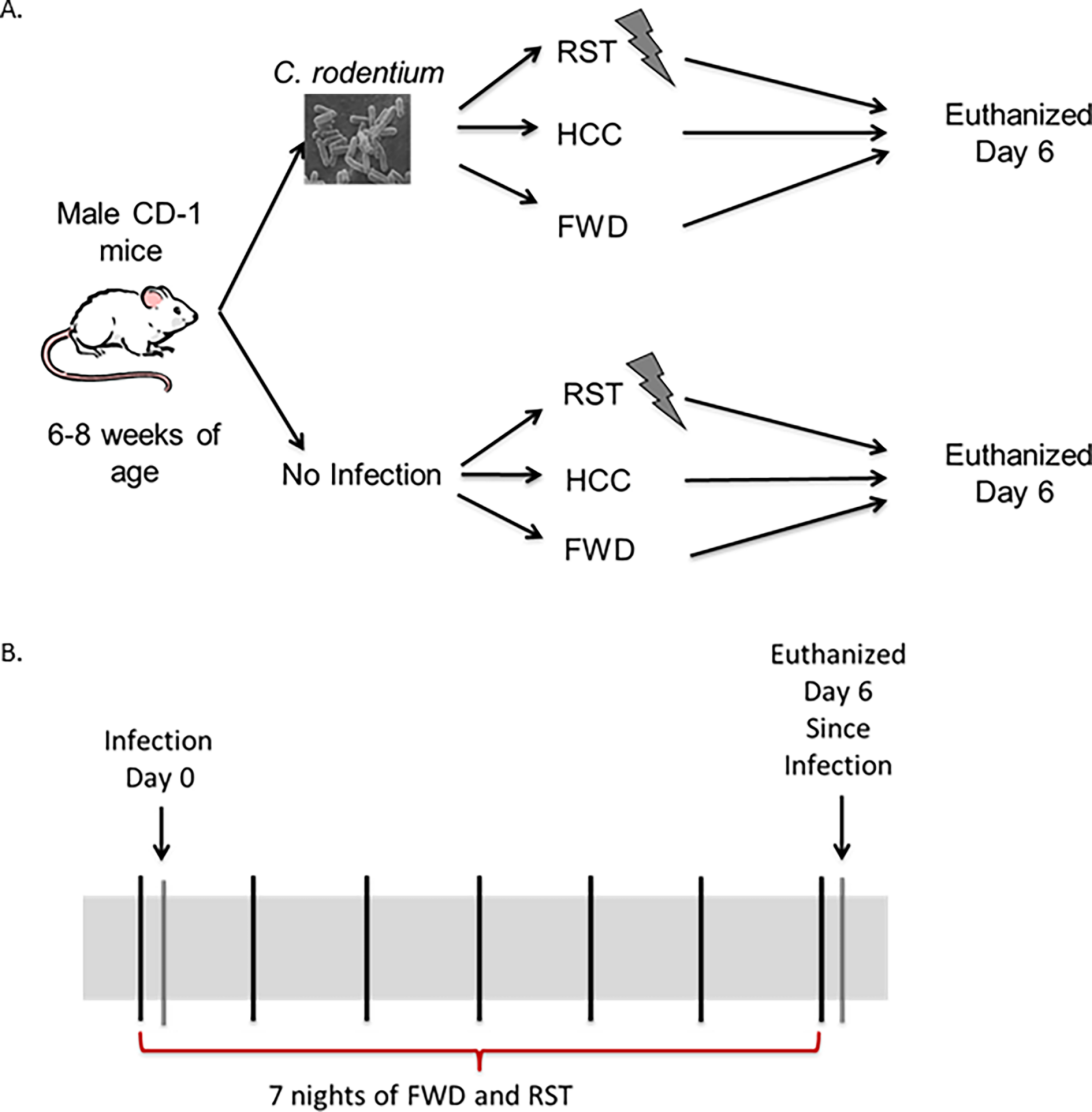
Experiment design. A. Half of the mice were challenged with the infection *Citrobacter rodentium by oral gavage*. Mice were then divided into three groups. Either exposed to the restraint (RST), food and water deprived (FWD), or home cage control (HCC). All of the mice were euthanized the morning after the 7^th^ night of RST. B. Mice exposed to RST were stressed 7 nights in a row. If mice were challenged with *Citrobacter rodentium* it occurred after the first episode of stressor exposure.

A total of 36 mice were divided into 6 different groups with 6 mice per group. One-third of the total mice were allowed to eat standard chow and water *ad libitum* throughout the entire experiment; this group was known as the home cage control group (HCC). One-third of the total mice were food and water deprived (FWD) during experimental procedures only, and 1/3 of the total mice were exposed to a prolonged restraint (RST) stressor and food and water deprived during stressor exposure 7 nights in a row. The RST stressor is conducted by placing the mice in a well ventilated 50 ml conical centrifuge tube with multiple holes for 14 hrs from 1800 to 0800. The FWD control group was only deprived of food and water for the same length of time as the RST group. The HCC group was not restrained or food and water deprived for the length of the experiment. After the first exposure to the stressor 1/2 of the total mice were challenged with *C*. *rodentium*. All mice were euthanized via compressed gas CO2 asphyxiation on day 6 post-challenge (Fig 1A and 1B) [2, 23]. Colonic tissue, cecum, luminal contents, spleens, and livers were collected, flash frozen in liquid nitrogen, and stored at -80°C.

### Citrobacter rodentium

*Citrobacter rodentium* strain DBS120(pCRP1::Tn5) was grown in Difco Lennox broth at 37°C overnight. Half of the mice were challenged with *C*. *rodentium* via oral gavage of 3x10^6^ CFU suspended in 100 μl PBS after the first round of RST. All data collected are referenced by the day post-challenge with *C*. *rodentium*, with day 0 as the initial day of infection. Fecal shedding of *C*. *rodentium* was determined via culture on day 6 post-challenge by plating stool on MacConkey agar with kanamycin (40ug/ml), in order to enumerate only *C*. *rodentium*. Each plate was incubated at 37°C with 5% CO₂ for 24 hours. The number of colonies were counted, and the colony forming units were calculated per gram of stool.

### Histopathology

Distal colons were placed in 10% formalin-buffered phosphate, embedded in paraffin, and stained with hematoxylin and eosin (H&E). Histopathology scoring was performed by a board certified veterinary pathologist who was blinded to treatment groups. Each section of colon was scored using a validated histopathology scoring system [39] that consisted of 5 different categories: inflammation, edema, hyperplasia, crypt atrophy, and epithelial defects. Each category was scored from a range of 0 to 4 in increments of 0.5. The degree of severity was as follows: 0, absent; 1, mild; 2, moderate; 3, marked; and 4, severe. A total histopathology score was calculated for each mouse ranging from 0 to 20.

### Semiquantitative real-time PCR

RNA was isolated from proximal colon via a standard single-step isolation protocol (TRIzol® Reagent; Life technologies, Carlsbad, CA) and cDNA synthesized (AMV RT kit; Promega, Madison, WI). RT-PCR was performed as previous published [2] on the ABI Prism 7000 system using primers targeting mRNA Table 1. Data was expressed as a fold-change from the uninfected, non-stressor exposed, non-FWD, day 6 control mice (HCC).

**Table 1:** Sequences of primers and probes used for real-time PCR.

Primer or probe	Primer direction	Sequence
**18s**	Forward	CGGCTACCACATCCAAGGAA
** **	Reverse	GCTGGAATTACCGCGGCT
** **	Probe	TGGCACCAGACTTGCCCTC
**TGFβ**	Forward	GCAACATGTGGAACTCTACCAGAA
** **	Reverse	GACGTCAAAAGACAGCCACTCA
**TNFα**	Forward	CTGTCTACTGAACTTCGGGGTGAT
** **	Reverse	CGGA(C/T)GTAAGGGCCGTGC
**IL-1β**	Forward	GGCCTCAAAGGAAAGAATCTATACC
** **	Reverse	GCTCTGGGCCATAGAACTGATG
** **	Probe	ATGAGAAGTTCCCAAATGGCCTCCCTC
**iNOS**	Forward	CAGCTGGGCTGTACAAACCTT
** **	Reverse	GTATTGCTTGGGATCCACACTCT
** **	Probe	ATGAAAGACGGCACACCCACCCTG
**GPR41**	Forward	GTGACCATGGGGACAAGCTTC
** **	Reverse	CCCTGGCTGTAGGTTGCATT
**GPR43**	Forward	GGCTTCTACAGCAGCATCTA
** **	Reverse	AAGCACACCAGGAAATTAAG
**GPR109A**	Forward	GGCGTGGTGCAGTGAGCA
** **	Reverse	CTGCAGGACTCTGTCTTCTCAGT

Primers and probes were purchased from (Thermo Fisher, Waltham, MA).

### Serum corticosterone levels

Corticosterone levels were measured in all groups. Corticosterone levels were assessed in serum and collected at the same time point when the mice were euthanized, which occurred after 7 days of stressor exposure. The samples were centrifuged at 5,000 g for 10 mins at 4°C to obtain serum, stored at -80°C until used. Serum corticosterone levels were analyzed using a commercial ELISA kit (Corticosterone ELISA kit; Abcam, Cambridge, MA) according to the manufacturer’s instructions.

### Short chain fatty acid assessment

Fecal samples were weighed and placed in a lyophilizer for 22 hrs. Dry fecal samples were suspended in 1 ml of 0.5% phosphoric acid per 0.1 g based on the wet fecal sample [40]. Samples were aggressively vortexed and centrifuge for 10 mins at 17,000 x g. The supernatant was separated from the fecal pellet and ethyl acetate was used at 1 ml for every ml of supernatant (1:1) as the extraction solvent. Samples were centrifuged again for 10 mins at 17,000 x g and the organic extract was removed and stored at -20°C [41].

#### GC-MS analysis

The gas chromatography-mass spectrometry (GC-MS) system consisted of a Trace Ultra GC equipped with an AS3000 auto-sampler coupled to a DSQ II mass spectrometer (Thermo Scientific, Waltham, MA). A stabliwax-DA (Restek, cat# 11023) highly polar column with polyethylene glycol stationary phase (PEG) was installed on the GC, and helium was used as the carrier gas at 1 mL/min. Split injection mode was used, with split flow and split ratio set at 10 and an injection temperature of 250°C. A blank solvent (ethyl acetate) was injected between every sample to ensure no memory effects. Each sample was injected twice to ensure consistency. A standard curve was generated for acetic acid, butyric acid, and propionic acid using 1000 μM, 800 μM, 400 μM, 200 μM, 100 μM, and 50 μM concentrations at the beginning of the run. A 400 μM standard (4-methylvaleric acid) was used throughout the runs to assess the consistency of results [41].

The initial column temperature was set at 90°C which was increased to 150°C at a rate of 15°C/min, then to 170°C at a rate of 5°C/min, and to 250°C at a rate of 20°C/min. A final temperature hold at 250°C for 2 min was used to clean the column. A solvent delay of 2.5 min was applied before collecting MS data in the *m/z* 50–150 range for 14 min. Electron ionization (EI) was used at 70 eV and the temperature of the ion source, sample inlet and MS transfer line were 230°C, 250°C and 240°C, respectively. Identification of differentially eluted SCFAs was based on varying retention times and the corresponding EI-MS spectra as compared to those in the NIST/EPA/NIH mass spectral libraries (searched using the NIST Mass Spectral Search Program v2.0, 2005) [41].

#### Processing method for SCFA calculated amount

The processing setup module in Xcalibur (Thermo Scientific, Waltham, MA) was used for rapid quantification of the absolute amount of SCFAs in each fecal sample using data obtained from standards at the start of the run. The target ions selected to generate extracted ion chromatograms and used for quantification of acetic acid, propionic acid, and butyric acid were *m/z* 60, 74, and 60 respectively. The ICIS peak detection algorithm was used to detect the highest peak in the expected retention time (RT) range for acetic acid (RT = 4.08, 20 second window), propionic acid (RT = 4.75, 20 second window), and butyric acid (RT = 5.53, 30 second window). The quality of the peaks used for quantitation was validated by enabling the resolution, symmetry and peak classification parameters. The calculated amounts of SCFAs were determined using the average of two injections of the same sample.

### 16S rRNA gene sequencing

QIAamp DNA minikit protocol (Qiagen, Valencia, CA) was used for DNA recovery from the mid-colonic section per manufacturer directions. DNA was quantified by using Qubit dsDNA broad range assay kit.

DNA was amplified targeting the V3-4 hypervariable region of the 16S rRNA gene (F: 5' TCGTCGGCAGCGTCAGATGTGTATAAGAGACAGCCTACGGGNGGCWGCAG; R:5’GTCTCGTGGGCTCGGAGATGTGTATAAGAGACAGGACTACHVGGGTATCTAATCC). Libraries were prepared (NexteraXT kit; Illumina) and equimolar samples pooled. Sequencing was performed via the Illumina MiSeq platform resulting in 10.6 million paired end reads (MiSeq Reagent Kit v3 600cycle; Illumina). Forward and reverse reads were merged using Quantitative Insights into Microbial Ecology (QIIME) version 1.9.1 with an overlap length of 40 and 95% similarity in the overlap region. Trimming and filtering at Q20 resulted in approximately 7 million reads. Trimmed and cleansed reads were loaded into QIIME. Closed reference OTU picking pipeline along with green genes dataset version 13.8 was used to produce OTUs incorporating 88% of the input reads. *De novo* OTU picking was performed using AbuandantOTU+ version 0.92b. AbuandantOTU+ incorporated 99% of the input reads after removing chimeric and contaminant OTUs. Based on Bokulich *et al* (2013), a taxa was retained if it had ≥ 0.005% of the total count [42]. Linear mixed effect model followed by ANOVA was conducted with Group and Infection as fixed effects and cage as a random effect [43]. For separate time points and pairwise comparisons, the model includes only the relevant terms. All P-values were False Discovery Rate (FDR) corrected. Alpha diversity was assessed using Chao1 and Shannon indexes using rarefied counts. Beta diversity was assessed using PCoAs designed from Bray-Curtis dissimilarity using Log 10 normalized counts according to the following formula:

$\log_{10}\left\langle \left(\frac{taxa\;raw\;count}{number\;of\;sequences\;in\;samples}\right)(average\;number\;of\;sequences\;persample)+1\right\rangle$ [43, 44]. This approach was also applied to the forward and the reverse reads independently without merging to validate the results from the merged reads.

### Statistical analysis

Standard two-way ANOVA were used to analyze *C*. *rodentium levels*, colonic IL-1β, IL-10, TNFα, TGFβ, iNOS, histopathology scores, SCFAs (acetic acid, propionic acid, and butyric acid), colonic SCFA receptors (GPR41, GPR43, and GPR109A), and serum corticosterone levels with stressor exposure (HCC, FWD, RST) and infection exposure (*C*. *rodentium* vs. vehicle) as the between subjects variables. An alpha level of *p* <0.05 was set as the rejection criteria for the null hypothesis. All data were analyzed using SPSS statistical software version 21 (IBM Corp.; Armonk, NY) and presented as treatment means ± standard error of the mean (SEM). Pearson’s correlation was used to identify significant associations between bacterial genera, SCFAs, and markers of intestinal inflammation. The Holm procedure was used for correction of multiple correlation analyses.

## Results

### Stressor exposure affected SCFAs and G protein coupled receptor expression

**Fig 2:**
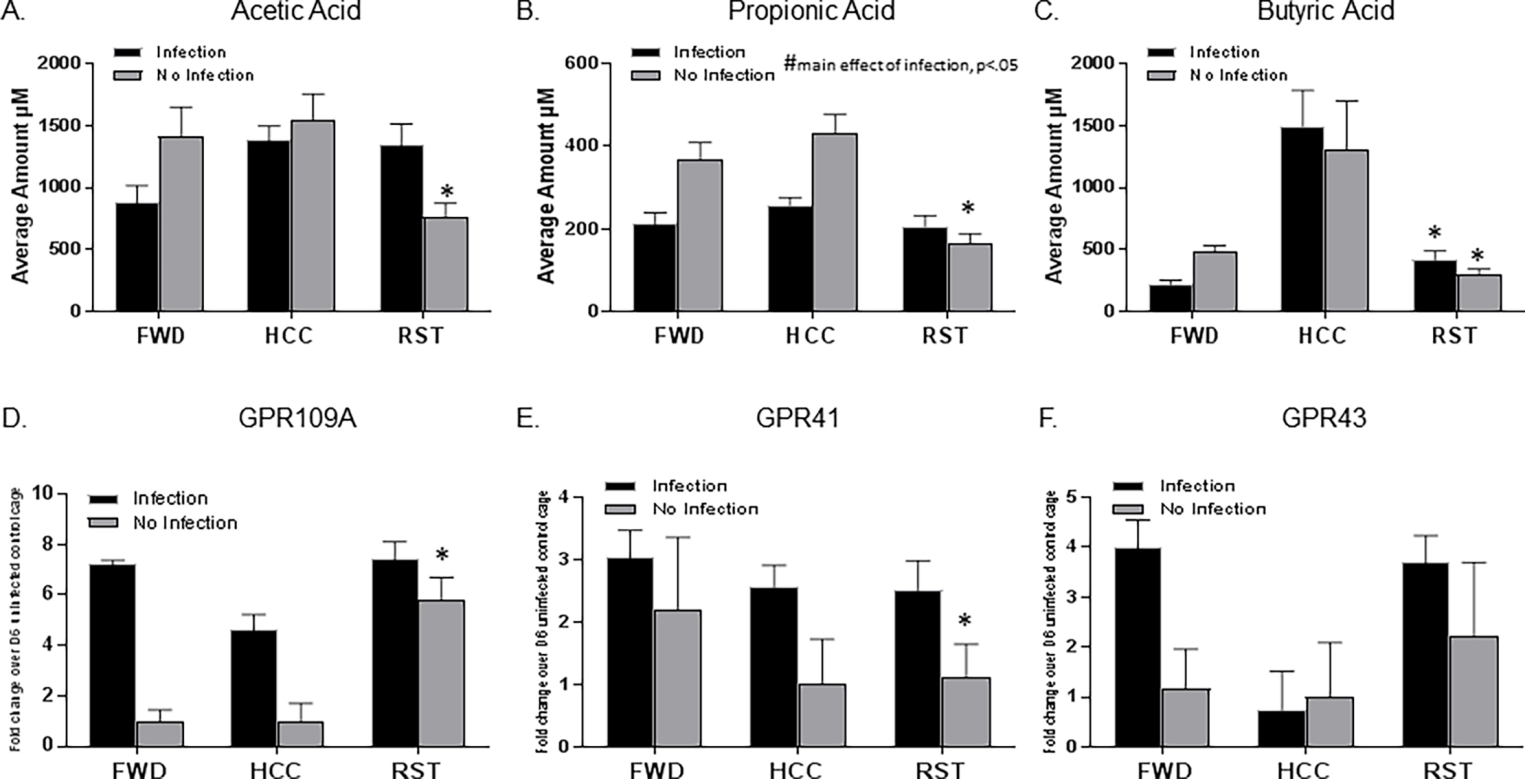
Stressor exposure decreases SCFA levels and increases the receptor expression of GPR109A. A. Acetic acid levels decreased with stressor exposure without infection (*p<0.05 vs HCC No Infection, FWD No Infection, HCC Infection, and RST Infection). B. Propionic acid levels decreased with stressor exposure without infection (*p<0.05 vs. HCC No Infection, FWD No Infection, and HCC Infection). Exposure to *C*. *rodentium* reduced propionic acid levels overall (#main effect of infection exposure, p<0.05). C. Butyric acid levels decreased with stressor exposure independent of infection (*main effect of stressor exposure, p<0.05). D. GPR109A receptor expression was increased in mice exposed to the stressor without infection (*p<0.05 vs HCC No Infection and FWD No Infection). E. Statistically significant interaction effect between stressor exposure and infection exposure on GPR41 receptor expression (*p<0.05, same stress exposure group but absence of infection). F. GPR43 receptor expression was not effected by stressor exposure or infection. Data are the mean +/- SEM.

Exposure to the prolonged RST stressor significantly reduced SCFA levels, but the effects of the stressor were dependent upon whether the animals were exposed to the infection. There was a significant interaction between stressor exposure and infection exposure on acetic acid levels in the two factor ANOVA (p<0.05). Post-hoc testing demonstrated that stressor exposure reduced acetic acid levels in uninfected mice, but not in mice challenged with *C*. *rodentium* (Fig 2A, p<0.05). There was also a significant interaction between stressor exposure and infection exposure for propionic acid levels (Fig 2B, p<0.05), with stressor exposure reducing propionic acid levels in the uninfected mice. This stressor-induced reduction in propionic acid was not evident in mice exposed to *C*. *rodentium*, but exposure to *C*. *rodentium* reduced propionic acid levels overall (i.e., main effect of infection exposure in the two factor ANOVA, p<0.05). Exposure to the stressor also reduced butyric acid levels in both infected and uninfected mice (main effect of stress, Fig 2C, p<0.05). Interestingly, post-hoc testing indicated that food and water deprivation (used as a control because stressor-exposed mice will not eat or drink in the restraining tubes) also reduced butyric acid levels (Fig 2C, p<0.05).

The expression of the G protein coupled receptors was also affected by stressor exposure. There was a significant interaction between stressor exposure and infection exposure on GPR109A expression (Fig 2D, p<0.05). Post-hoc analyses indicated that stressor exposure significantly increased GPR109A levels in uninfected mice, but this stressor-induced increase in GPR109A did not occur in mice challenged with *C*. *rodentium*. There was a trend for *C*. *rodentium* challenge to increase GPR109A expression, but this was not statistically significant (Fig 2D, p = 0.07). There was a statistically significant interaction effect between stressor exposure and infection exposure on GPR41 expression (Fig 2E, p<0.05), but post-hoc testing indicated that this was due to significant increases in GPR41 in HCC and RST mice (p<0.05), but not FWD mice, when challenged with *C*. *rodentium*. Neither stressor exposure nor infection exposure affected GPR43 expression (Fig 2F) as indicated by the lack of significant main effects or an interaction effect in the two factor ANOVA.

### Stressor exposure and *C*. *rodentium* challenge altered the composition of the gut microbiota

**Fig 3:**
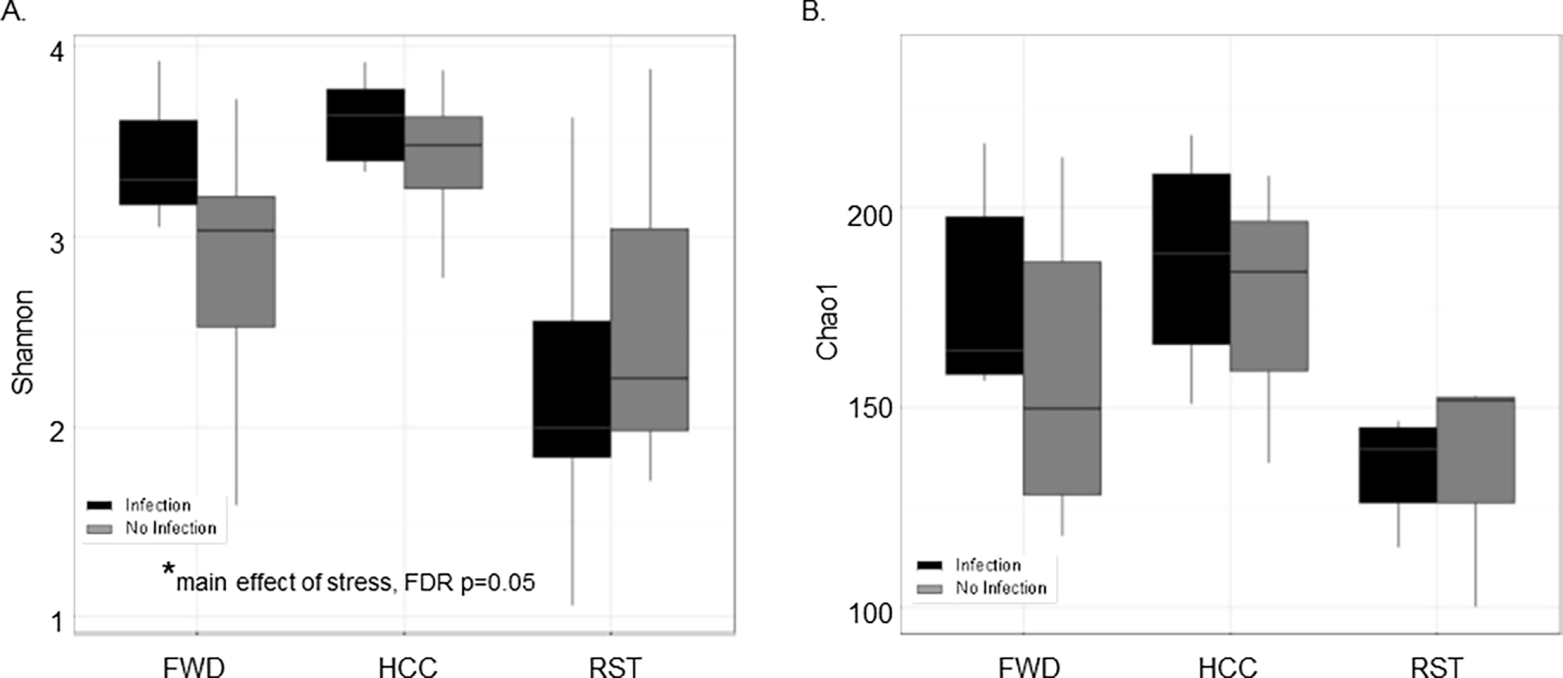
Mice exposed to the restraint stressor had a decrease in alpha diversity *de novo*. A. Shannon diversity significantly decreased with stressor exposure (*main effect of stressor exposure, FDR p = 0.05). B. Chao1 diversity showed a trend with a decrease in diversity with stressor exposure (FDR p = 0.067). Data are presented as the median (line), interquartile range (box), and minimum and maximum (whiskers).

Short chain fatty acids are produced by the commensal microbiota, thus the composition of the microbiota was analyzed to verify that stressor exposure changed the composition of the gut microbiota. Alpha diversity measures the richness and evenness of species within a sample, and was evaluated with two different indexes, the Shannon diversity index (SDI) and the Chao 1 index. The SDI evaluates diversity based on operational taxonomic unit (OUT) distribution and abundance in a sample, whereas the Chao 1 index takes into account the number of rare classes to evaluate the total richness of the sample. The SDI was significantly different between the groups (HCC, FWD, and RST) (Fig 3A, FDR p = 0.05). The stressor-exposed group had a significantly decreased SDI in comparison to the FWD and HCC group indicating a decrease in richness and evenness of the community. There was a tendency for mice exposed to the infection to have, a decreased Chao 1, but the difference was not statistically significant between the groups (Fig 3B, FDR p = 0.067).

**Fig 4:**
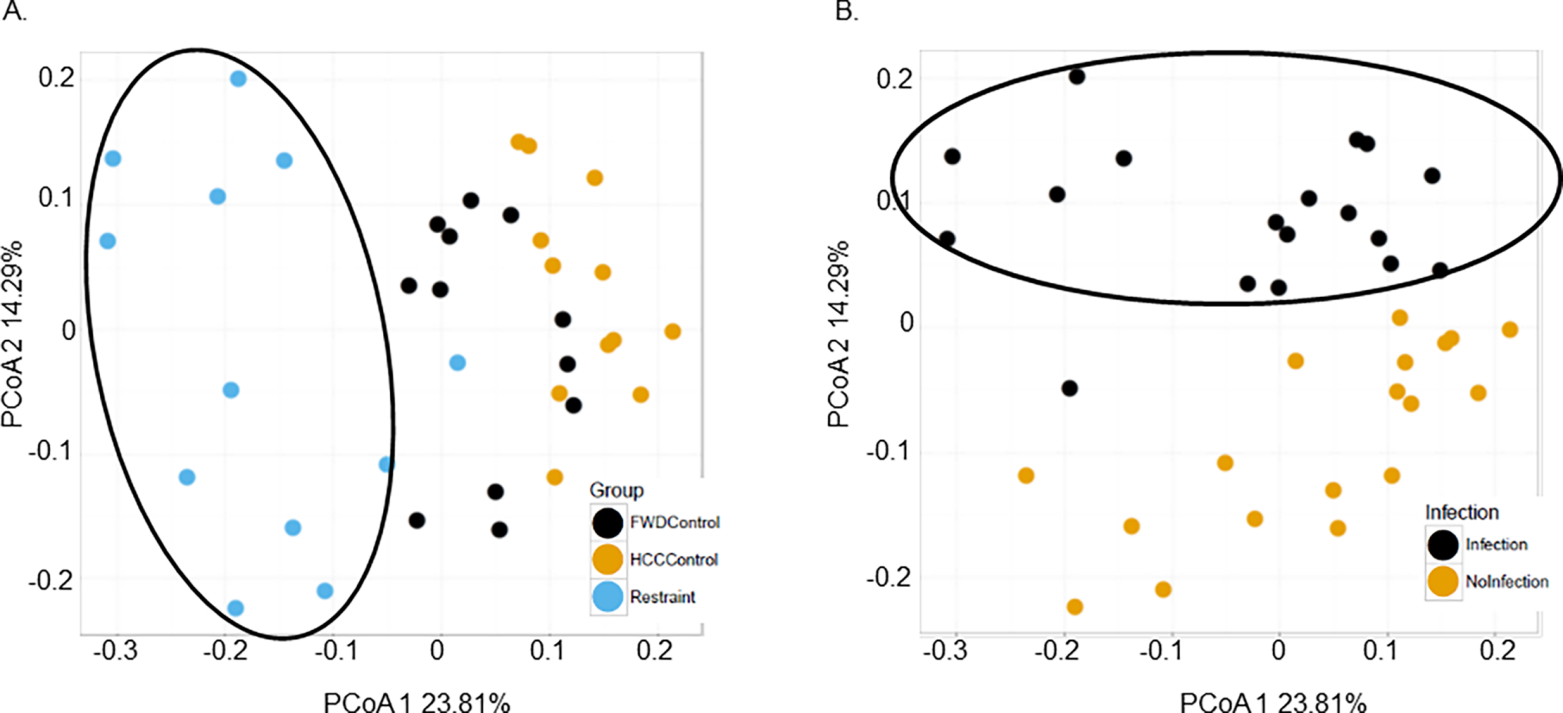
Beta diversity using QIIME closed reference OTUs was significantly affected by stressor exposure and when challenged by the infection. A. Mice exposed to the RST stressor (depicted with a black circle around) clustered separately from the HCC group and the FWD group. The FWD group also clustered separately from the HCC group and RST group. The PCoA 1 and 2 scores were significantly different between groups (FDR p<0.05). B. Mice exposed to the infection (depicted with a black circle around) clustered separately from samples from mice not exposed to the infection (FDR p<0.05 on PCoA axis 2).

Beta diversity measures the difference in OTU composition between different samples and was assessed using principal coordinate analysis (PCoA) generated from Bray-Curtis dissimilarity at the OUT level generated using QIIME closed reference OTUs. The RST stressor, FWD, and HCC groups all clustered separately on a PCoA plot with statistically significant differences on PCoA axis 1 and 2 (Fig 4A, FDR p<0.05). Samples separated by whether they were exposed to the infection on PCoA axis 2 (Fig 4B, FDR p<0.05). Parallel analysis using linear mixed effect model analysis also showed statistically significant difference between the RST, HCC, and FWD groups on PCoA axis 1 and 3, and between infection and no infection on PCoA axis 3 (Data not shown, FDR p<0.05).

**Fig 5:**
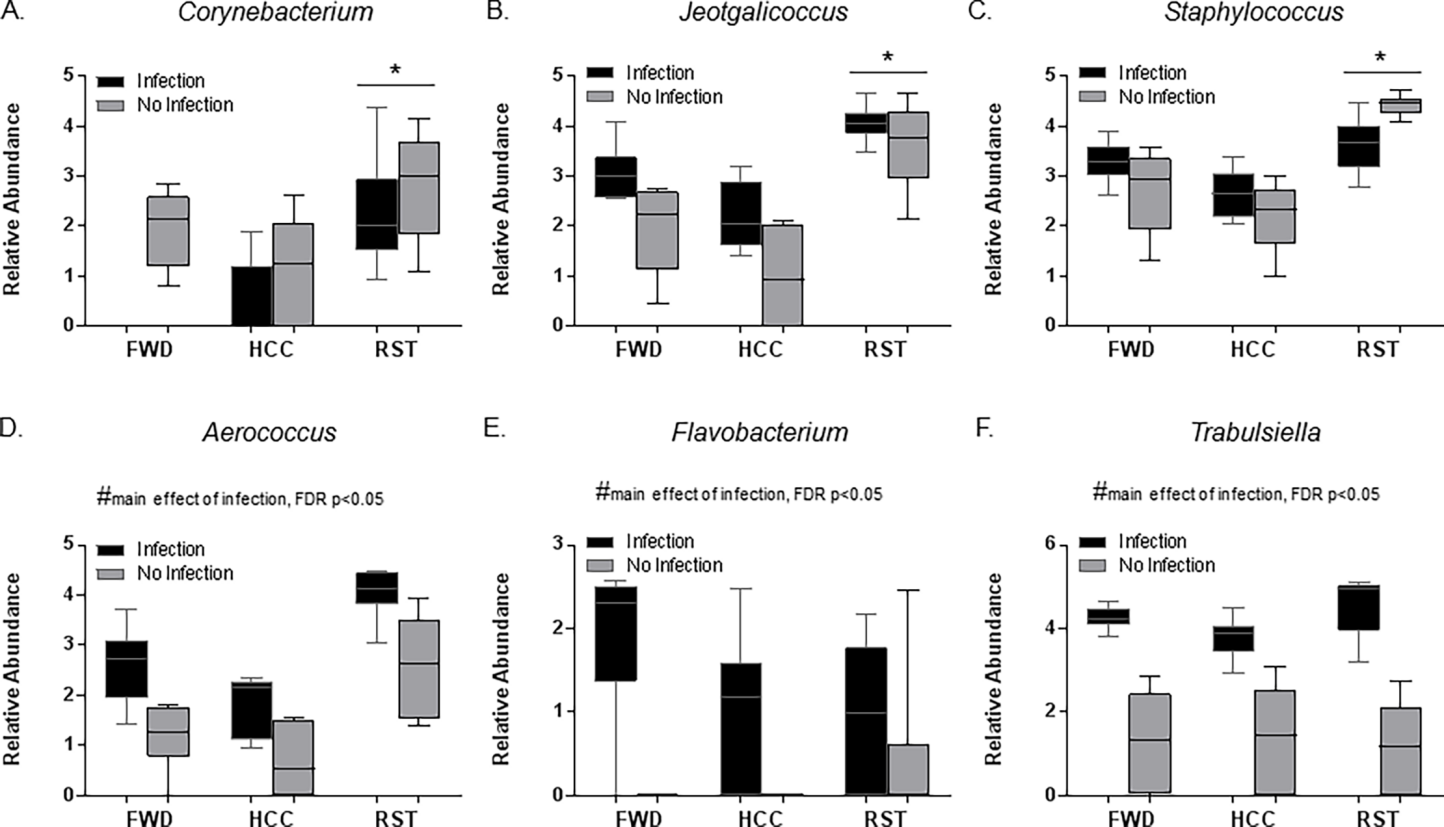
Stressor exposure and *C*. *rodentium* challenge altered the composition of the gut microbiota. The relative abundance of multiple taxa were affected by stressor exposure and/or *C*. *rodentium* challenge, including A. *Corynebacterium* (*FDR p<0.05 vs HCC). B. *Jeotgalicoccus* (*FDR p<0.05 vs HCC). C. S*taphylococcus* (*FDR p<0.05 vs HCC). D. *Aerococcus* (#main effect of infection, FDR p<0.05). E. *Flavobacterium* (#main effect of infection, FDR p<0.05). F. *Trabulsiella* (#main effect of infection, FDR p<0.05). Data are presented as the median (line), interquartile range (box), and minimum and maximum (whiskers).

**Fig 6:**
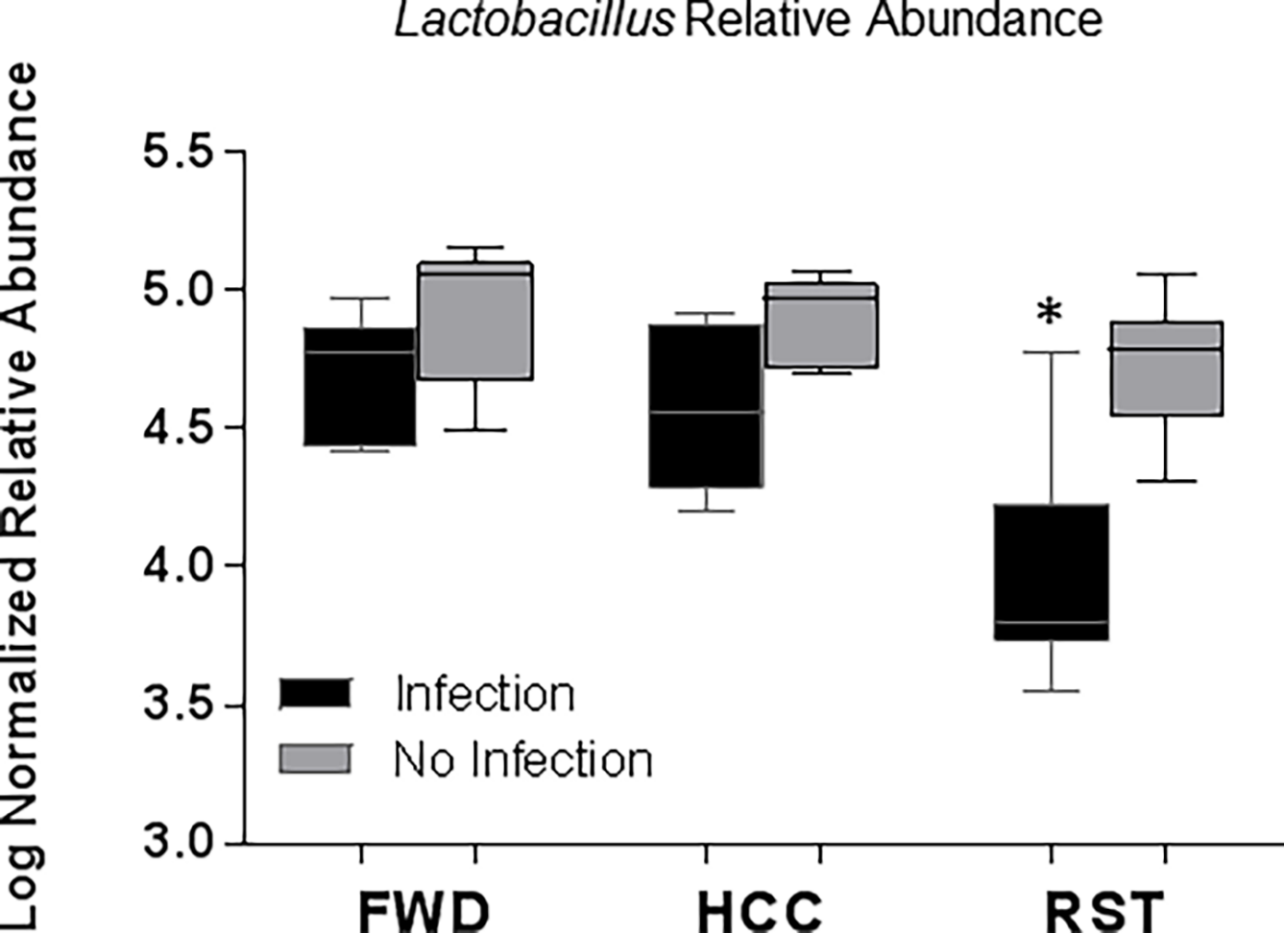
*Lactobacillus* relative abundance was affected by stressor exposure. *Lactobacillus* relative abundance was significantly reduced when exposed to the stressor and challenged with *C*. *rodentium* (*p<0.05 vs. HCC No Infection, FWD No Infection, RST No Infection, HCC Infection, and FWD Infection). Data are presented as the median (line), interquartile range (box), and minimum and maximum (whiskers).

Taxonomic analysis at the genus level showed that stressor exposure and infection exposure had significant effects between RST, FWD, and HCC groups. The relative abundances of *Corynebacterium*, *Jeotgalicoccus*, *and Staphylococcus* were significantly increased with RST stressor exposure vs HCC, but not vs FWD (Fig 5A–5C, FDR p<0.05). Infection exposure caused an increase in the relative abundance of *Trabulsiella*, *Flavobacterium*, and *Aerococcus* in all the groups (Fig 5D–5F, FDR p<0.05). We and others have previously published that stressor exposure affects the relative abundance of *Lactobacillus*, [18, 20–22, 45]. Thus, we tested our a priori hypothesis that stressor exposure would change the relative abundance of *Lactobacillus*. When stressor exposed mice were challenged with *C*. *rodentium*, there was a significant reduction in *Lactobacilli* relative abundance (Fig 6, p<0.05). In the absence of infection, the mean *Lactobacillus* relative abundance in stressor-exposed animals was lower than in control mice, but this difference did not reach statistical significance.

### The restraint stressor significantly increases *C*. *rodentium* levels in the lumen of the colon

**Fig 7:**
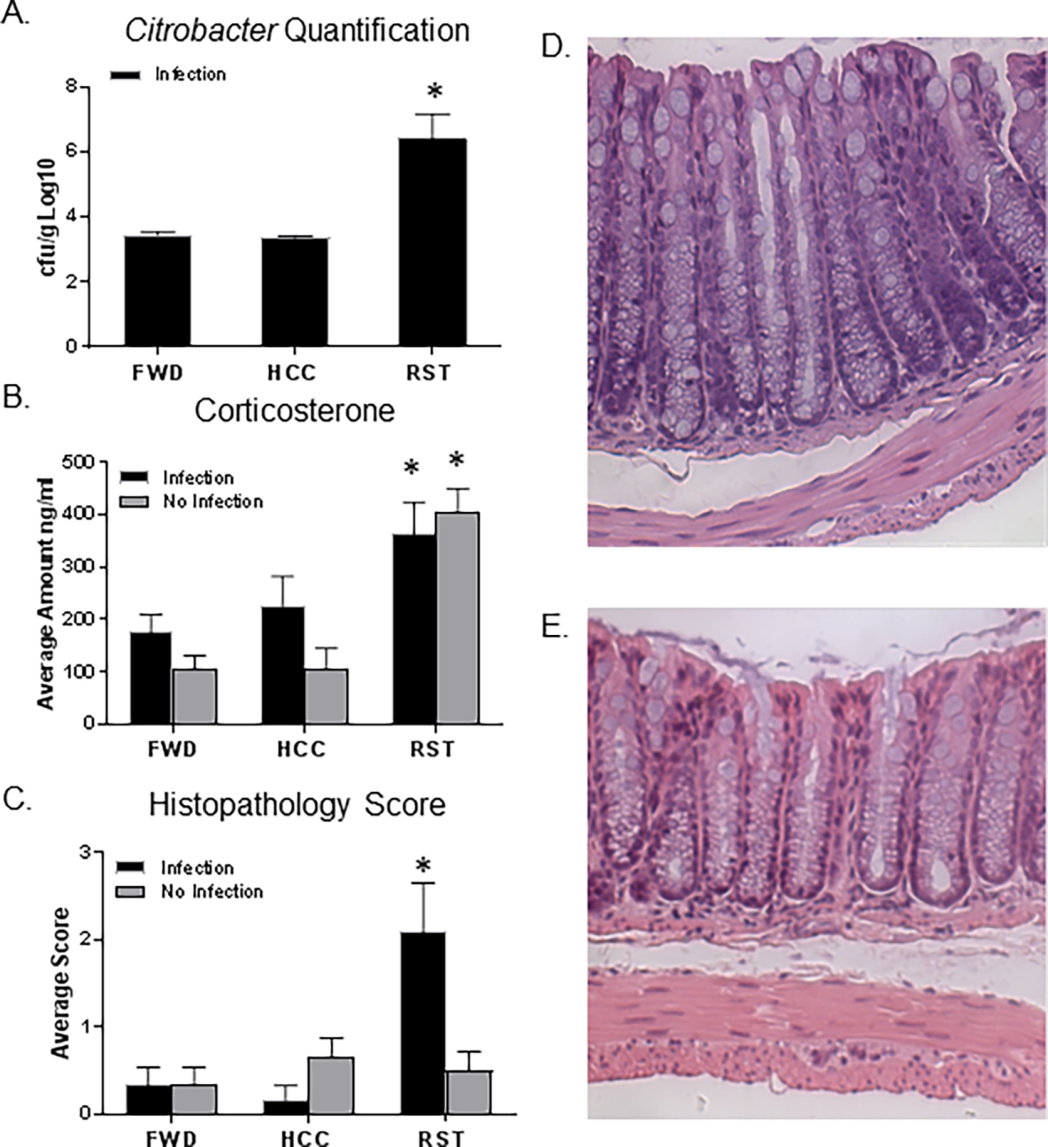
Stressor exposure increases *C*. *rodentium* levels and colonic histopathology scores. A. *Citrobacter* levels significantly increased in feces of stressor exposed mice (*p<0.05, vs HCC Infection and FWD Infection). B. Corticosterone levels were significantly increased in mice exposed to the RST stressor (*p<0.05, main effect of stress). C. Mice exposed to the infection and exposed to the RST stressor had a significant increase in histopathology scores (*p<0.05, vs HCC No Infection, FWD No Infection, RST No Infection, HCC Infection, and FWD Infection). D. A representative image of the histopathology for a mouse with mild disease exposed to the infection and RST stressor. E. A representative image of the histopathology for a mouse with no inflammation. All representative histologic sections are from the distal colon and are shown at the same magnification of 20X. Data are the mean +/- SEM.

Exposure to the stressor significantly increased *C*. *rodentium* levels in the stool by approximately 1000 fold (Fig 7A, p<0.05). Mice that were challenged with *C*. *rodentium*, but were not exposed to the stressor, had lower levels of *C*. *rodentium* 6 days post-challenge that just exceeded the limit of detection of our assays (approximately 1x10^3^ CFU/g). Mice in the FWD control condition did not have significant increases in *C*. *rodentium* levels (Fig 7A).

### Exposure to the stressor increased corticosterone levels

Corticosterone levels were significantly increased in mice exposed to the RST stressor regardless if they were exposed to the infection, representing a significant stressor-induced increase in corticosterone (Fig 7B, P<0.05).

### Exposure to the stressor increased *C*. *rodentium*-induced colonic histopathology

Mice exposed to the infection and RST stressor had an average colonic histopathology score of 2.1 out of 20, which is a mild degree of colonic inflammation (Fig 7C). Although mild, this group had a significantly higher histopathology score in comparison to all other groups (p<0.05). In the absence of the RST stressor, *C*. *rodentium* did not significantly increase histopathology scores in HCC or FWD groups. Increases in *C*. *rodentium*-induced colonic histopathology were only observed in animals that were also exposed to the RST stressor. Representative histologic sections showing mild inflammation (pathology score of 3.5) and normal histology (pathology score of 0) are provided (Fig 7D and 7E, respectively).

### Stressor exposure and *C*. *rodentium* significantly increased inflammatory markers in the colon

**Fig 8:**
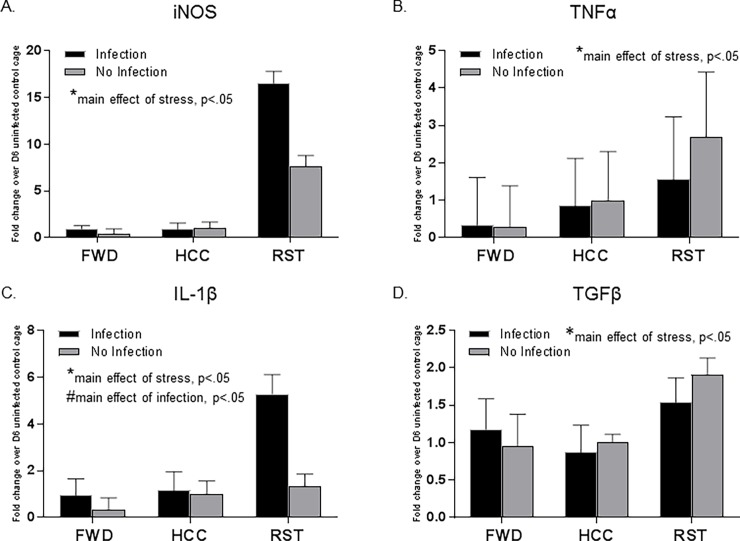
Restraint stressor exposure and *C*. *rodentium* significantly increased inflammatory markers in the colon. A. qPCR analysis stressor exposure significantly increased iNOS expression (*main effect of stressor exposure, p<0.05). B. qPCR analysis stressor exposure significantly increased TNFα expression (*main effect of stressor exposure, p<0.05). C. qPCR analysis stressor exposure and infection exposure significantly increased IL-1β expression (*main effect of stressor exposure, p<0.05) (#main effect of infection exposure, p<0.05). D. qPCR analysis stressor exposure significantly increased TGFβ expression (*main effect of stressor exposure, p<0.05). Data are the mean +/- SEM.

Mice exposed to the RST stressor had significant increases in the expression of inflammatory cytokines iNOS, TNFα, and IL-1β and an increase in anti-inflammatory cytokine TGFβ (Fig 8A–8D) (main effect of stress, p<0.05). Mice exposed to the infection also had a significant increase in the expression of IL-1β (main effect of infection, p<0.05).

### The gut microbiota and SCFAs are directly associated with stressor-induced increases in intestinal inflammation

To determine whether the microbiota were associated with changes in tissue inflammation and SCFA levels, the relative abundances of the genera were used in correlation analyses. *Lactobacillus* had a significant negative association (p<0.001) with GPR109A. *Aerococcus* demonstrated a significant positive association with iNOS, IL-1β, and histopathology scores (p<0.001), but a negative trend with propionic acid (p<0.05). *Lactobacillus* had a significant negative association with IL-1β (p<0.001) and a negative trend with iNOS (p<0.01). *Lactobacillus* was also significantly positively associated with propionic acid (p<0.001). *Lactobacillus* was not significantly associated with histopathology scores, but had a negative trend (p = 0.09).

## Discussion

This study confirms and extends results from our previous studies that have shown that the RST stressor changes the composition of the gut microbiota and leads to heightened colonic inflammation after pathogen challenge [2, 17, 18]. Importantly, this study further demonstrates that the RST stressor significantly affects the levels of SCFA’s in the gut. There was a decrease in acetic acid, propionic acid, and butyric acid with prolonged RST stressor exposure, which is consistent with observations in inbred C57BL/6 mice exposed to an acute social stressor (Maltz et al., Manuscript Under Review). *C*. *rodentium* challenge also decreased propionic acid levels, whereas *C*. *rodentium* did not significantly affect butyric acid or acetic acid levels. The lack of a pathogen effect on butyric acid and acetic acid was unexpected. However, a decrease in SCFAs right after stressor exposure, and at the time of pathogen challenge, could set the stage for the enhanced inflammatory process that was evident in stressor-exposed mice, since SCFAs have been used to reduce inflammation [24, 46].

Stressor exposure also caused an increase in the expression of the SCFA receptor GPR109A in mice that were not challenged with the colonic pathogen. There was also a trend for pathogen challenge to increase GPR109A. The expression of GPR109A has been reported to be induced by the gut microbiota [32, 33]. In our study, the relative abundance of *Lactobacillus* was inversely correlated with GPR109A. GPR109A has been shown to reduce intestinal inflammation in animal models [34], thus the tendency for GPR109A expression to be increased with stress and infection, and to be positively correlated with markers of intestinal inflammation, was unexpected. However, the only known ligands for GPR109A are butyrate and niacin [32, 33, 47, 48]. Butyrate levels in stressor-exposed mice were significantly reduced in the current study. It is not known if niacin was also reduced by stressor exposure, but endogenous levels of niacin are typically too low to activate GPR109A which has a low affinity for niacin [32, 33, 47, 48]. Thus, although GPR109A expression was increased, the ligands for the receptor were reduced in stressor-exposed mice, thus decreasing the likelihood that the GPR109A receptor was actually activated.

GPR41 and GPR43 were not significantly reduced by stressor exposure, nor where they significantly associated with colonic inflammation. However, propionic acid which can bind to both GPR41 and GPR43 [49, 50], was reduced in mice when exposed to the stressor and challenged with *C*. *rodentium*. Activation of GPR41 and GPR43 has been shown to reduce colonic inflammatory responses [27, 30, 51], and in the current study propionic acid levels were inversely correlated with markers of colonic inflammation. This suggests that although GPR41 and GPR43 expression was not changed, reduced activation of these receptors due to low levels of propionic acid may have contributed to stressor-induced increases in colonic inflammation. Such a role for propionic acid and GPR41/GPR43 requires further studying.

Although not assessed in the current study, SCFAs can also affect colonic inflammatory responses through their effects on histone deacetylases (HDAC) [52–54]. In particular, butyrate and propionate are well recognized to inhibit class I HDACs [32, 34]. Severe colonic inflammation is often associated with overexpression of HDAC, leading to epigenetic modifications in the colon [55, 56]. Inhibition of HDAC through SCFAs can reduce epigenetic modifications, and in turn reduce inflammation. Thus, future studies should assess whether epigenetic modifications are associated with the observed stressor-enhanced colonic inflammatory responses.

It is possible that SCFAs, as well as their receptors, were not directly involved in stressor-enhanced colonic inflammation, but instead reflected dysbiosis in the colon. We have shown that alpha and beta diversity were significantly affected by the RST stressor [18], but whether pathogen challenge further affects the microbiota of restrained mice has not been previously tested. In previous studies, stressor-induced changes in the microbiota were found to lead to increased severity of colonic inflammation in pathogen-challenged mice; germ-free mice colonized with microbiota from stressor-exposed mice had increased colonic inflammation upon pathogen challenge [17]. The current study has confirmed results from previous studies that stressor exposure, and now stressor exposure with pathogen challenge, reduces the relative abundance of bacteria in the genus *Lactobacillus*. Stressor-induced reductions in *Lactobacillus* have been found in mice, nonhuman primates, as well as humans [4, 18, 20–22, 45]. Reductions in *Lactobacillus* could be contributing to increased colonic inflammation during stressor exposure, since previous studies have found that administering probiotic *Lactobacillus* ameliorates colonic inflammation, including iNOS and TNFα [4, 23, 57–60]. *Lactobacillus* given as a probiotic in a DSS model of inflammation also led to a decrease in clinical scores, histopathology, and inflammatory cytokines (IFNγ, TNFα, IL-6, and IL-1β) [58]. In the current study, lower relative abundances of commensal lactobacilli were significantly correlated with higher expression of IL-1β (with trends for being correlated with higher iNOS). In support of a potential protective role of propionic acid, *Lactobacillus* relative abundances were directly correlated with propionic acid levels; propionic acid levels were low when lactobacilli levels were low. This is perhaps not surprising since *Lactobacillus* spp. are prolific producers of lactic acid that can be readily converted to propionic acid [61]. However, further work is needed to understand the relationship between low lactobacilli and stressor-enhanced colonic inflammation.

When considered together, this study shows that exposure to the RST stressor leads to an increase in corticosterone and leads to changes in microbial community alpha and beta diversity, with relative abundances of specific genera (primarily *Aerococcus* and *Lactobacillus*) being associated with the onset of pathogen-induced colonic inflammation and with SCFA levels. It is possible that alterations in SCFAs, including butyric acid and propionic acid, are involved in stressor-induced increases in colonic inflammatory responses, since stressor and pathogen exposure changed SCFA levels. *Lactobacillus* was inversely correlated with GPR109A expression, and when activated, GPR109A can reduce colonic inflammation. However, the primary ligand for GPR109A (i.e., butyric acid) was significantly reduced in this study, suggesting that increased GPR109A would not reduce inflammatory responses. *Lactobacillus* directly correlated with propionic acid, which can have anti-inflammatory effects when acting through GPR41 or GPR43. Both *Lactobacillus* and propionic acid were inversely associated with colonic inflammation, supporting the contention that *Lactobacillus*-associated propionic acid helps to regulate the mucosal inflammatory response. These results offer some insight to better understand interactions between gut microbiota and colonic inflammatory responses during stressful periods.
